# Older African Immigrants’ Experiences of Discrimination in the United States

**DOI:** 10.1093/geroni/igaa057.2408

**Published:** 2020-12-16

**Authors:** Manka Nkimbeng, Janiece Taylor, Laken Roberts, Peter Winch, Yvonne Commodore-Mensah, Roland Thorpe, Hae-Ra Han, Sarah Szanton

**Affiliations:** 1 University of Minnesota, Minneapolis, Minnesota, United States; 2 Johns Hopkins School of Nursing, Baltimore, Maryland, United States; 3 Johns Hopkins University School of Nursing, Baltimore, Maryland, United States; 4 Johns Hopkins Bloomberg School of Public Health, Baltimore, Maryland, United States; 5 Johns Hopkins University, Baltimore, Maryland, United States

## Abstract

Discrimination is implicated in the disproportionate burden of disease and health disparities in racial/ethnic minorities. This qualitative descriptive study explored the experiences of discrimination and its impact on the health of older African immigrants. Semi-structured interviews were conducted with 15 participants. Three main themes and six sub-themes were identified. These included: 1) types of discrimination: a) accent-based, b) unfair treatment during routine activities, c) experience with police and other systems; 2) costs of discrimination; 3) surviving and thriving with discrimination: a) “blind eye to it”, b) reacting to it, c) avoiding it. These themes describe common forms of discrimination that these older adults have experienced, current strategies used to deal with discrimination, and the impact of discrimination on the wellbeing of this sample. To improve the emotional and mental health of older African immigrants, providers serving them should assess for perceived discrimination, and refer participants with any concerns for treatment.

